# The impact of platelet-rich plasma injection on anterior cruciate ligament reconstruction: a systematic review and meta-analysis

**DOI:** 10.3389/fbioe.2025.1625271

**Published:** 2025-10-01

**Authors:** Yiran Zhang, Zhikang Xiao, Zhe Fan, Yingxin Zhang, Jianzhong Xu, Kun Wang

**Affiliations:** ^1^ Department of Orthopedics, Fourth Medical Center of Chinese PLA General Hospital, Beijing, China; ^2^ College of Medicine, Zhengzhou University, Zhengzhou, Henan, China; ^3^ Department of Orthopedics, The First Affiliated Hospital of Zhengzhou University, Zhengzhou, Henan, China

**Keywords:** platelet-rich-plasma, anterior cruciate ligament reconstruction, knee, systematic review, meta-analysis

## Abstract

**Purpose:**

This systematic review and meta-analysis evaluates platelet-rich plasma (PRP) efficacy in anterior cruciate ligament reconstruction (ACLR) through 15-year Randomized controlled trials (RCTs) data, focusing on postoperative recovery, rehabilitation acceleration, and functional outcomes optimization.

**Methods:**

We conducted an extensive systematic search in PubMed, Embase, and Web of Science to find relevant studies on using PRP in ACLR. Randomized controlled trials analyzing the comparative effectiveness of PRP compared to control interventions in individuals undergoing ACLR were systematically identified. The focus was on studies that provided reliable outcome measures, encompassing validated clinical assessments and objective imaging results. Outcome indicators included the Visual Analog Scale (VAS) for pain perception, the International Knee Documentation Committee (IKDC) score, Lysholm score, Tegner activity scale, KT-1000 side-to-side difference, graft characteristics, and associated complications. Additionally, subgroup analyses were categorized based on evaluation timelines, distinguishing between preoperative and postoperative assessments.

**Results:**

This meta-analysis of 24 studies demonstrated time-dependent effects of PRP supplementation following ACLR. The PRP group exhibited significant improvement in IKDC scores at 12 months post-operatively (mean difference: 2.09, P = 0.01, I^2^ = 23%), while Lysholm scores showed significant enhancement at 6 months (mean difference: 3.33, P = 0.03, I^2^ = 58%). Pain reduction, assessed by VAS scores, was significantly greater in the PRP group at 3 months (mean difference: −1.33, P < 0.01, I^2^ = 38%) with borderline significance at 6 months (mean difference: −0.78, P = 0.05). Notably, PRP intervention significantly reduced anterior tibial translation compared to controls (mean difference: −1.34 mm, 95% CI: −1.56 to −1.13, P < 0.01, I^2^ = 73%), indicating improved knee stability. Pre-operative KT-1000 measurements suggested a trend toward reduced knee laxity in the PRP group (mean difference: −0.70 mm, 95% CI: −1.45 to 0.05, P = 0.07), though this effect did not persist post-operatively. No significant between-group differences were observed in Tegner activity scores, Signal-to-Noise Quotient, or Pivot Shift Test results at any follow-up interval.

**Conclusion:**

This meta-analysis indicates that PRP application during and shortly after ACLR offers limited clinical benefits. Although there is notable short-term pain relief, long-term efficacy remains unclear, with improvements not meeting minimal clinically important differences (MCID) and no significant changes in knee stability or graft maturation. Further research is needed to establish optimal PRP protocols and standardization for ACLR.

## 1 Introduction

This study systematically reviewed randomized controlled trials from the past 15 years that met the criteria for analysis, incorporating relevant outcome data for a meta-analysis. The purpose was to analyze and evaluate the clinical and graft outcomes at different postoperative intervals after applying Platelet-Rich Plasma (PRP) in Anterior cruciate ligament reconstruction (ACLR), while also examining its impact on accelerating sports rehabilitation and facilitating an early return to exercise, thereby providing guidance for clinicians. We hypothesize that the combined application of PRP and ACLR could enhance knee function and promote graft healing.

ACLR is a surgical technique designed to restore the knee joint’s stability and functionality. The procedure consists of replacing the torn ligament completely with a tissue graft, providing a biological structure that assists in the regeneration and integration of the ligament (Bailey et al., 2021). ACLR is a widely adopted treatment for ACL injuries in physically active individuals experiencing knee instability. Although primary ACLR generally yields favorable outcomes, the clinical results for patients undergoing revision surgery following reinjury are consistently inferior, highlighting the need for improved strategies in secondary interventions. According to studies, the failure rate of revision ACLR is three to four times that of primary ACLR (Marois et al., 2022; Guy et al., 2022). Of those who undergo revision ACLR, 40.2% are able to resume their prior activity level, while 34.7% have not returned to sports after an average of 9 years of follow-up (Cristiani et al., 2021). In this context, PRP therapy has emerged as a potential adjunct treatment, garnering attention for its ability to enhance healing and reduce complications following ACLR. PRP involves extracting autologous blood, processing it to concentrate platelets, and injecting it at the surgical site to facilitate tissue repair via the release of growth factors. However, the efficacy of PRP in improving postoperative outcomes and mitigating re-injury risks remains debated. While some studies reveal promising results indicating improved recovery times and graft stability (Kia et al., 2018), others fail to establish a consistent benefit, raising questions about its routine application in clinical practice (Davey et al., 2020).

Over the previous 15 years, extensive research involving animals and clinical studies has focused on the use of PRP for ACLR. Preclinical research has consistently shown that PRP can enhance tissue repair and promote healing in injured or degenerative ACLs, supporting its potential clinical application (Hammad, 2020). However, in the context of human ACL injury repair, the combined use of PRP with ACL reconstruction has yielded inconsistent clinical outcomes. Kishor Munde and colleagues demonstrated that PRP application enhances graft healing at the tunnel interface and improves functional scores at 6 months postoperatively, highlighting its potential benefits in specific aspects of recovery (Bailey et al., 2021; Komzák et al., 2015). Likewise, research by Rong-jin Chen et al. showed that PRP aids in the tendon-bone healing of ACL grafts and enhances joint function shortly after surgery (Chen et al., 2022). Conversely, a study by Zipeng Ye and colleagues found that three intra-articular injections of PRP postoperatively did not significantly improve knee symptoms or function in the long term (Ye et al., 2024).

In addition to PRP, several other adjuvant therapies have been explored in conjunction with ACLR to enhance recovery and optimize clinical outcomes. Notably, corticosteroid injections have been investigated for their anti-inflammatory properties, potentially reducing postoperative pain and improving function in the early phases of rehabilitation (Puzzitiello et al., 2022). Additionally, hyaluronic acid injections aim to enhance joint lubrication and promote healing within the synovial fluid environment, thereby facilitating smoother joint movement and reducing the likelihood of complications (Arrich et al., 2005). Biological scaffolds and mesenchymal stem cell therapies are emerging as promising adjuncts as well, providing structural support and promoting tissue regeneration (Kang et al., 2021). Despite the availability of these therapies, PRP stands out due to its ability to concentrate growth factors and cytokines that actively promote healing and tissue repair (Wu X. et al., 2017). The unique biological mechanisms of PRP not only facilitate immediate recovery but also support long-term joint health. Therefore, this study aims to evaluate the efficacy of PRP in ACLR, highlighting its distinct advantages over other adjunct therapies and emphasizing its role as a vital component in the rehabilitation process.

## 2 Methods

The review was conducted in accordance with the Cochrane Handbook for Systematic Reviews of Interventions, ensuring the use of rigorous methods and standardized protocols.

### 2.1 Search strategy

By 24 July 2024, a systematic search of the PubMed, Embase, and Web of Science databases was carried out by researchers. A comprehensive search for relevant studies was conducted using a combination of the following terms and their variations, appearing in the title, abstract, or keywords. The search expressions included:

• (‘Anterior Cruciate Ligament Reconstruction’ OR ‘Anterior Cruciate Ligament Tear’ OR ‘Anterior Cruciate Ligament’ OR ‘ACL injury’ OR ‘ACL tear')
• AND (‘Platelet-Rich Plasma’ OR ‘Platelet Rich Plasma’ OR ‘PRP’ OR ‘Plasma’ OR ‘Platelet-Rich').

The detailed search expressions and results can be found in Supplementary Table. Detailed Search Strategies and Database Results.

### 2.2 Eligibility criteria for study selection

Inclusion criteria:

• Utilizing PRP during or post-ACL reconstruction surgery;
• RCTs that have been released in publications;
• Outcome measures included at least one of the following clinical results: VAS for pain, IKDC score, Lysholm score, Tegner score, KT-1000 measurement, pivot shift test, tunnel widening (assessed via computed tomography [CT]), graft characteristics (evaluated by magnetic resonance imaging [MRI]), and complications;
• Articles published primarily in English or Chinese;
• Complete data for outcome measures.

Exclusion criteria:

• Review papers, abstracts from conferences, case studies, and other types of non-original research articles;
• Interventions that did not include the use of PRP;
• Incomplete experiments or studies without reported results;
• Data that is incomplete, unclear, or contains obvious errors, and cannot be resolved by contacting the authors.

### 2.3 Literature screening and data extraction

An initial literature review was performed based on set inclusion and exclusion criteria, and a secondary evaluation was done to remove duplicate articles. To ensure the integrity and precision of the selected studies, a thorough verification process involving full-text examination was undertaken. Each article was meticulously evaluated to confirm its eligibility and alignment with the research objectives. In conclusion, 24 studies met the inclusion criteria. The meta-analysis gathered information on the essential characteristics of the chosen studies, which comprised the following details: first author, publication year, country, follow-up period, assessment timelines, sample size, age range of participants, and gender distribution. Furthermore, specifics regarding the PRP intervention, including the number of injections, volume [mL], injection timing, graft type, and centrifugation parameters, were cataloged. Primary outcomes focused on VAS pain scores, IKDC scores, and Lysholm scores, whereas secondary outcomes encompassed the Tegner score, KT-1000 arthrometer laxity measurement, drawer test, tunnel widening, and graft characteristics.

Literature screening for this study was conducted by Yiran Zhang, who identified relevant studies based on predefined inclusion and exclusion criteria. The data extraction process was performed by Zhikang Xiao, who systematically recorded key characteristics of each included study. In the event of discrepancies during data extraction, Author Zhe Fan served as an arbitrator to ensure data consistency and accuracy. Any disagreements were resolved based on the study’s objectives, ensuring a coherent approach to result reporting.

### 2.4 Methodologic quality assessment

The potential for bias will be systematically assessed in seven domains across 24 randomized controlled trials (RCTs), as per the Cochrane Risk of Bias Assessment Tool’s criteria (Higgins and Green, 2011). These domains include: Random Sequence Generation (Selection Bias), Allocation Concealment (Selection Bias), Blinding of Participants and Personnel (Performance Bias), Blinding of Outcome Assessment (Detection Bias), Incomplete Outcome Data (Attrition Bias), Selective Reporting (Reporting Bias), and Other Bias. Each study will be assessed and categorized as having a Low Risk, High Risk, or Unclear Risk of bias for each domain. The studies’ overall quality will be assessed by combining risk evaluations from all areas, resulting in a classification of high, moderate, or low quality. Specifically, if all studies are assigned a ‘Low Risk’ rating across all domains, the cumulative bias risk is regarded as low, indicating high research quality. In cases where one or two studies are rated as ‘High Risk’ or ‘Unclear Risk,’ the research will be classified as having a moderate risk of bias. Conversely, if more than two studies are rated ‘High Risk,’ the overall bias risk will be classified as high, suggesting a potential compromise in the integrity of the research findings.

### 2.5 Data synthesis

The data primarily comprises continuous and dichotomous variables. Averages and standard deviations are used to report continuous variables, while dichotomous variables are given in absolute values. For each category of outcome measures, all variables maintain consistent units of measurement. Each result includes its calculated original mean difference (MD) and 95% confidence interval (CI). Heterogeneity among variables is evaluated using a forest plot, with the degree of heterogeneity determined by the I^2^ statistic. A fixed-effects model is used for analysis if heterogeneity is determined to be statistically insignificant (I^2^ < 50%). Conversely, a random-effects model is used when there is significant heterogeneity (I^2^ ≥ 50%). To further investigate the relationship between PRP application and postoperative recovery time and efficacy, subgroup analyses are performed to compare scores from the VAS, Lysholm, and IKDC at different time points. All results are analyzed using Review Manager Version 5.3.

## 3 Results

### 3.1 Study screening and selection

**FIGURE 1:**
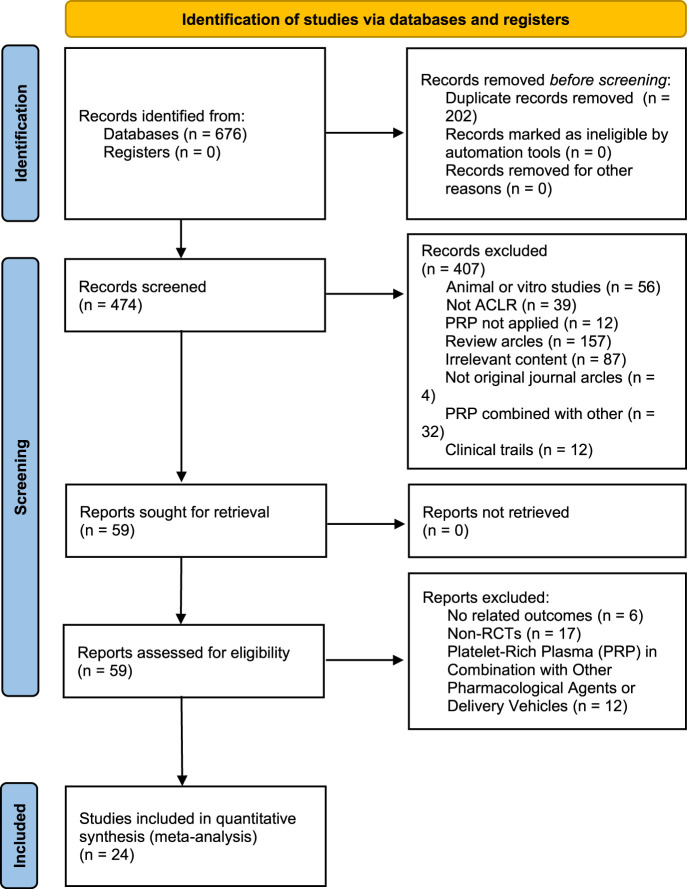
PRISMA (Preferred Reporting Items for Systematic Reviews and Meta-Analyses) study selection flow diagram.

A total of 676 studies were retrieved from three databases: PubMed, Web of Science, and Embase. From this collection, 32 studies were identified as RCTs. In the end, 24 of these RCTs satisfied the predetermined inclusion criteria and were incorporated into the data analysis, as depicted in the flowchart in Figure 1.

### 3.2 Study quality

**FIGURE 2:**
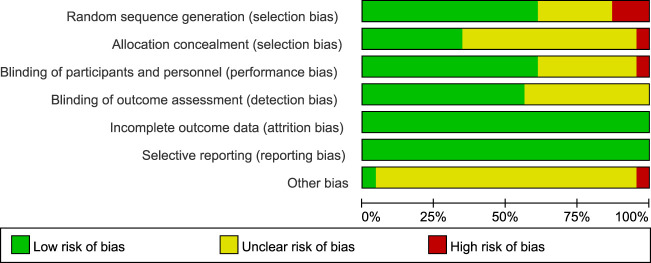
Details of the study quality assessment according to Cochrane Collaboration risk-of-bias tool.

**FIGURE 3:**
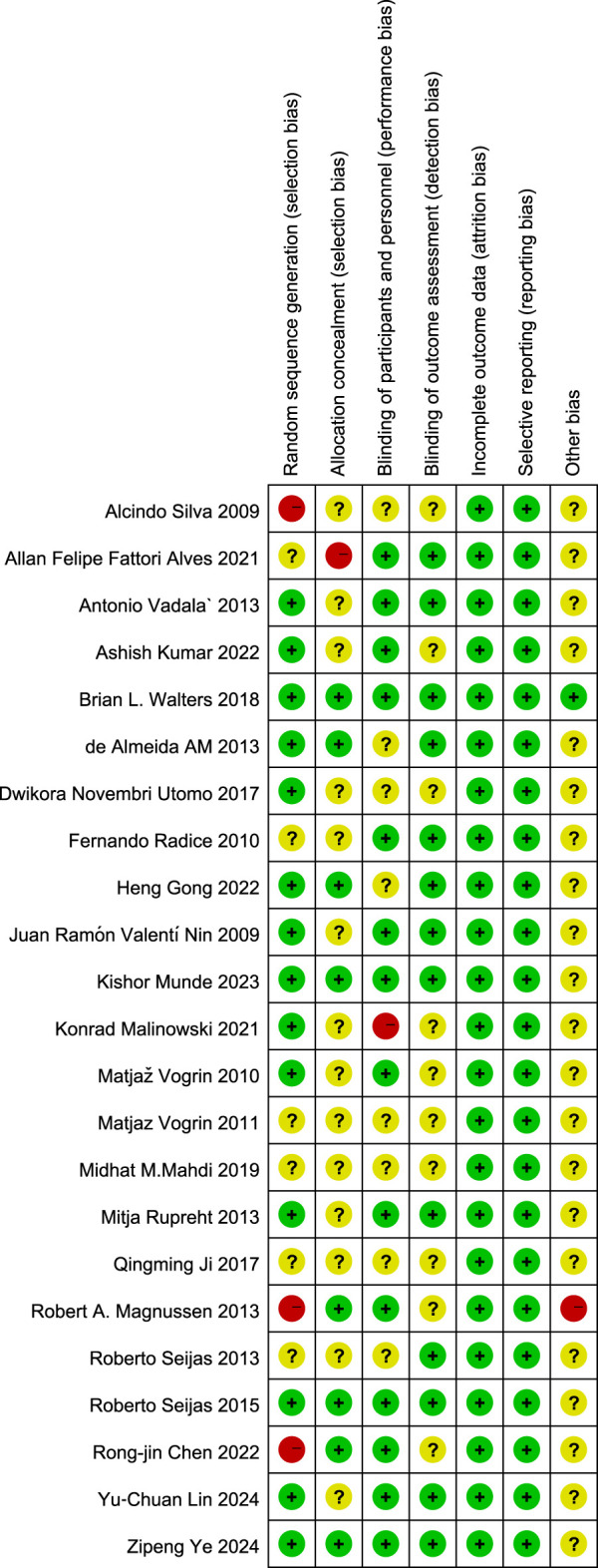
Details of the study quality assessment according to Cochrane Collaboration risk-of-bias tool.

Risk-of-bias appraisal with the Cochrane RoB 2.0 tool (Figures 2, 3) indicates a generally robust evidence base. Seventeen of the 24 RCTs (70.8%) were judged low risk across all seven domains, five trials (20.8%) were rated high risk owing mainly to inadequately reported allocation concealment or random sequence generation, and two trials (8.4%) attracted “some concerns” in a single domain. Appropriate sequence generation, blinding of participants, personnel, and outcome assessors, and complete outcome reporting were documented in more than four-fifths of studies, whereas clear descriptions of allocation concealment were provided by only about half. No study showed high risk for attrition bias or selective reporting, and the few unclear judgements for “other bias” largely reflected incomplete disclosure of funding sources. Sensitivity analyses that excluded the five high-risk trials yielded effect estimates virtually identical to those of the primary analyses, underscoring the stability of the review’s findings.

### 3.3 Study characteristics

The characteristics of the included studies are summarized in Table 1. A total of 1,411 patients participated in the study, assigned to either the PRP group or the control group. Ages of participants spanned from 14 to 65 years, averaging around 31 years. Follow-up durations varied between 3 months and 2 years, averaging around 12 months. Assessments were primarily conducted at 3-, 6-, and 12-month post-operation. The specifics regarding PRP administration, including dosages, are presented in Table 2. The dosage of PRP ranged from 0.8 to 40 mL, with an average of 7.1 mL. Except for the trials conducted by Ye Z. (Ye et al., 2024), Ji Q (Ji et al., 2017). and Silva A. (Silva and Sampaio, 2009), all other studies administered PRP as a single dose during the operation.

**TABLE 1:** Characteristics of studies included.

First Author	Year	Country	Patients, n	Sex, M/F, n	Age, y, mean ± SD (range)	Follow-up Period (range)	Assessment Time (range)
Yu-Chuan Lin	2024	China	27	17/10	PRP = 28.4 ± 7.8 non = 29.7 ± 9.9	48 weeks	Pre-op and 12, 24, 48 wk post-op
Zipeng Ye	2024	China	120	84/36	29.0 ± 8.0	12 months	Pre-op and 3, 6, 12 mo post-op
Kishor Munde	2023	India	87	74/6	PRP = 28.37 ± 2.8 non = 27.40 ± 7.0	6 months	6 mo
Ashish Kumar	2022	India	70	45/25	PRP = 28.34 ± 4.32 non = 29.71 ± 2.99	3 months	Pre-op and im, 6, 12 wk post-op
Heng Gong	2022	China	60	39/21	PRP = 33.5 ± 8.97 non = 34.9 ± 9.68	12 months	Pre-op and 3, 6, 12 mo post-op
Rong-jin Chen	2022	China	85	49/36	PRP = 32.01 ± 11.23 non = 35.90 ± 10.31	12 months	Pre-op and 3, 6, 12 mo post-op
Konrad Malinowski	2021	Poland	106	NR	NR	18 months	im and 18 mo post-op
Allan Felipe Fattori Alves	2021	Brazil	34	19/15	32.0 ± 7.0	3 months	Pre-op and 3 mo post-op
Midhat M.Mahdi	2019	Iraq	27	27/0	25.77	12 weeks	12 wk post-op
Brian L. Walters	2018	USA	50	22/28	30.0 ± 12.0	2 years	12 wk, 6 mo, 1 and 2 years post-op
Dwikora Novembri Utomo	2017	Indonesia	20	NR	NR (20–35)	14 months (8–20 mo)	14 mo (8–20 mo)
Qingming Ji	2017	China	42	15/21	PRP = 31.59 non = 33.68	16 months	Pre-op and 3, 12 mo post-op
Roberto Seijas	2015	Germany	43	NR	NR (18–65)	96 weeks	1, 2, 4, 6, 9, 12 mo post-op
Antonio Vadala`	2013	Italy	40	40/0	34.5 (18–48)	14.7 months (10–16 mo)	Pre-op and 14.7 mo (10–16 mo) post-op
Roberto Seijas	2013	Spain	98	NR	NR (18–65)	12 months	4, 6, 12 mo post-op
Mitja Rupreht	2013	Slovenia	50	NR	PRP = 37.2 ± 8.4 non = 32.6 ± 12.3	6 months	1, 2.5, 6 mo post-op
First Author	Year	Country	Patients, n	Sex, M/F, n	Age, y, mean ± SD (range)	Follow-up Period (range)	Assessment Time (range)
de Almeida AM	2013	Brazil	27	24/3	24.3	6 months	6 mo post-op
Robert A. Magnussen	2013	USA	100	56/44	PRP = 35.1 ± 11.3 non = 35.3 ± 11.5	2 years	10 ± 4 days and 8 ± 4 wk post-op
Matjaz Vogrin	2011	Slovenia	50	NR	NR	26 weeks (25–27 wk)	5 (4–6 wk), 11 (10–12 wk), 26 (25–27 wk) wk post-op
Laura De Girolamo	2011	Italy	40	NR	NR	12 months	12 mo post-op
Matjaž Vogrin	2010	Slovenia	45	30/15	PRP = 35.4 ± 10.0 non = 33.0 ± 12.5	6 months	Pre-op and 3, 6 mo post-op
Fernando Radice	2010	Chile	50	39/11	NR (18–35)	12 months	3, 9, 12 mo post-op
Alcindo Silva	2009	Portugal	40	38/2	26.8 ± 5.3	3 months	3 mo post-op
Juan Ramón Valentí Nin	2009	Spain	100	78/22	PRP = 26.1 (14–57) non = 26.6 (15–59)	2 years	Pre-op and 3, 6, 12 mo post-op

F, female; M, male; post-op, postoperative; pre-op, preoperative; mo, month; PRP, platelet-rich plasma (group); non, contral group; NR, no record.

**TABLE 2:** PRP injection characteristics of the included studies.

First Author (Year)	Dosing Frequency	Total PRP Dose	Volume of Autologous Blood for PRP (anticoagulant)	Timing of Injection	Injection Site	Graft Type
Yu-Chuan Lin (2024)	1	3.5 mL	10 mL (NR)	in-op, post-op	applied at both ends of the graft and injected into the knee joint	Semitendinosus and gracilis tendons
Zipeng Ye (2024)	3	5/5/5 mL	45 mL (5 mL)	at 4, 8 wk and 3 mo post-op	the patellar tendon and toward the intercondylar notch	Semitendinosus and gracilis tendon
Kishor Munde (2023)	1	3–4 mL	17 mL (NR)	in-op	femoral tunnel	Semitendinosus and gracilis tendon
Ashish Kumar (2022)	1	15 mL	NR	post-op (immediate)	graft, femoral and tibial tunnels	Hamstring graft
Heng Gong (2022)	1	4 mL	36 mL (4 mL)	in-op (before the graft was pulled into the bone tunnels for fixation)	bone tunnels and graft	Semitendinosus and gracilis tendons
Rong-jin Chen (2022)	1	4.5–5 mL	8 mL (NR)	post-op	semitendinosus harvest site	Hamstring tendon
Konrad Malinowski (2021)	1	NR	NR	in-op (before and after graft insertion)	the graft harvest site, onto the intra-tunnel portions of the graft	Auadriceps tendon-bone full thickness autograft
Allan Felipe Fattori Alves (2021)	1	4 mL	NR	in-op (at the end)	in loco	Semitendinosus and gracilis tendons
Midhat M.Mahdi (2019)	1	6 mL	100–150 mL (NR)	in-op (at the end)	femoral tunnel	Semitendinosus and gracilis tendons
Brian L. Walters (2018)	1	3–5 mL	10 mL (1 mL)	in-op (before the initiation of closing and after fixation of the graft)	patellar donor site	BPTB autograft
Dwikora Novembri Utomo (2017)	1	0.8 mL	10 mL (0.5 mL)	post-op	knee joint	NR
First Author (Year)	Dosing Frequency	Total PRP Dose	Volume of Autologous Blood for PRP (anticoagulant)	Timing of Injection	Injection Site	Graft Type
Qingming Ji (2017)	3	10/5/5 mL	100/50/50 mL (NR)	in-op, 15 and 30 days post-op	articular injection	Semitendinosus and gracilis tendons
Roberto Seijas (2015)	1	4 mL	NR	post-op (immediate)	patellar bone gap, tibial bone gap, harvest gap, tendinous area	Patellar graft
Antonio Vadala’ (2013)	1	15 mL	10 mL (NR)	in-op (before passing the graft through the femoral tunnel)	between the peripheral part of the graft and the tunnel wall, above the graft	Semitendinosus and gracilis tendons
Roberto Seijas (2013)	1	8 mL	NR	post-op (immediate)	suprapatellar joint	BPTB autograft
Mitja Rupreht (2013)	1	5 mL	NR	in-op	femoral and tibial tunnels, graft itself	Semitendinosus and gracilis tendons
de Almeida AM (2013)	1	20–40 mL	NR	in-op	patellar tendon	BPTB autograft
Robert A. Magnussen (2013)	1	NR	NR	in-op	intra-articular portion of the graft	Tibialis tendons
Matjaz Vogrin (2011)	1	NR	NR	NR	NR	NR
Laura De Girolamo (2011)	1	NR	60 mL (NR)	NR	the femoral and the tibial level of the patellar tendon harvesting site	BPTB autograft
Matjaž Vogrin (2010)	1	6 mL	52 mL (8 mL)	in-op	femoral and tibial tunnels and into the graft itself	BPTB autograft
Fernando Radice (2010)	1	5 mL	60 mL (NR)	in-op (at the moment of inoculation on the graft)	on the graft	Hamstring tendon
Alcindo Silva (2009)	1 or 3	1.5 mL/time	27 mL (3 mL)	end in-op, at im, 2, 4 wk post-op	femoral tunnels	Semitendinosus and gracilis tendons
Juan Ramón Valentí Nin (2009)	1	4 mL	40 mL (NR)	in-op	ligament and tibial tunnel	BPTB allograft

ACL, anterior cruciate ligament; ACLR, ACL, reconstruction; BPTB, bone–patellar tendon–bone; C, control; NR, not reported in the original paper; PRGF, plasma rich in growth factors; PRP, platelet-rich plasma.

### 3.4 Objective assessment of joint stability

#### 3.4.1 KT-1000 side-to-side difference

**FIGURE 4:**
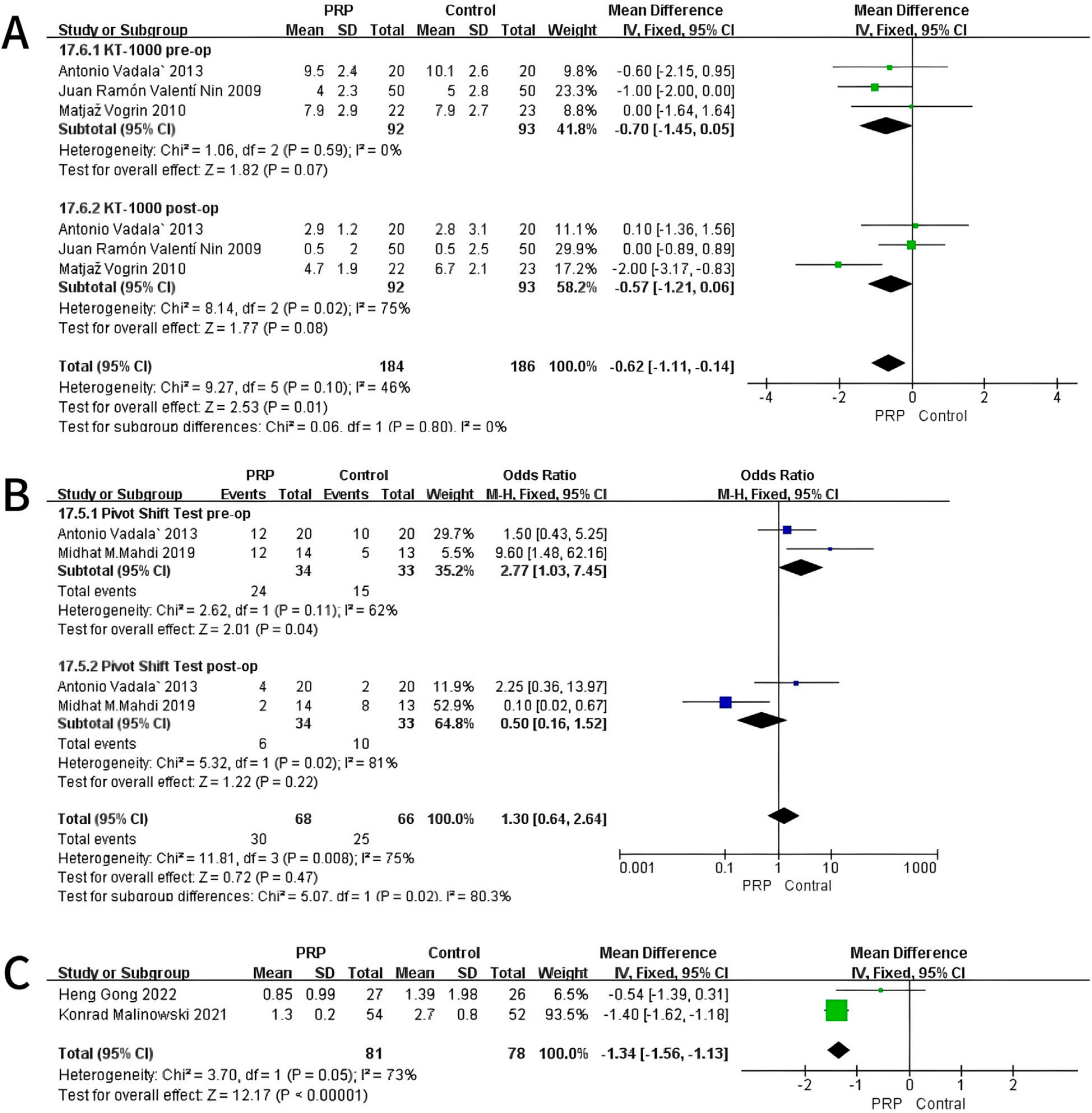
Forest Plot of KT-1000 measurement **(A)** and Pivot-shift Test at baseline and final follow-up after intraoperative application of PRP **(B)**. And Anterior Tibial Translation at 12 months after intraoperative application of PRP **(C)**.

A fixed-effects meta-analysis was performed on the KT-1000 measurements to compare the PRP group with the Control group. Preoperative analysis involving three studies (n = 185) revealed a mean difference of −0.70 mm (95% CI: −1.45 to 0.05) in favor of the PRP group. However, this result was not statistically significant (P = 0.07) and showed low heterogeneity (I^2^ = 0%, P = 0.59). In the postoperative assessment, the same studies indicated a mean difference of −0.57 mm (95% CI: −1.21 to 0.06), again favoring the PRP cohort, but without reaching statistical significance (P = 0.08), presenting moderate heterogeneity (I^2^ = 75%, P = 0.02). At all evaluated time points, the study did not find any statistically significant differences between the PRP and Control groups. These findings suggest a potential trend toward enhanced knee stability in the PRP group, which warrants further investigation (Figure 4A).

#### 3.4.2 Pivot-shift test

A fixed-effects meta-analysis was conducted on Pivot-Shift Test comparing PRP and Control groups. The pre-surgical analysis of two studies with 67 subjects indicated an odds ratio of 2.77 (95% CI: 1.03–7.45) supporting the Control group, with statistical significance (P = 0.04) and moderate heterogeneity (I^2^ = 62%, P = 0.11). Post-operation studies indicated an odds ratio of 0.50 (95% CI: 0.16–1.52) in favor of PRP, though it was not statistically significant (P = 0.22), with high heterogeneity (I^2^ = 81%, P = 0.02). The analyses revealed a statistically significant difference pre-operatively favoring the Control group. No significant difference was observed post-operatively. These results indicate a potential shift in Pivot-Shift Test outcomes from pre-to post-operative stages (Figure 4B).

#### 3.4.3 Anterior tibial translation (ATT)

A fixed-effects meta-analysis was conducted on ATT measurements comparing the PRP and Control groups. The review encompasses two studies (n = 138) indicating a mean difference of −1.34 mm (95% CI: −1.56 to −1.13), favoring the Control group. This difference was statistically significant (P < 0.01), with moderate heterogeneity (I^2^ = 73%, P = 0.05), indicating variability in the results across the included studies. As shown in Figure 4C, the individual studies contributed as follows: Heng Gong 2022 reported a mean difference of −0.54 mm (95% CI: −1.39 to 0.31) with a weight of 6.5%, while Konrad Malinowski 2021 showed a mean difference of −1.40 mm (95% CI: −1.62 to −1.18) with a weight of 93.5%. The overall mean difference across both studies is represented in the forest plot, demonstrating a significant reduction in ATT measurements for the PRP group compared to controls. The homogeneity of the results and moderate heterogeneity further support the reliability of the meta-analysis (Figure 4C).

### 3.5 Comprehensive evaluation of clinical outcomes

#### 3.5.1 International knee documentation committee (IKDC) score

**FIGURE 5:**
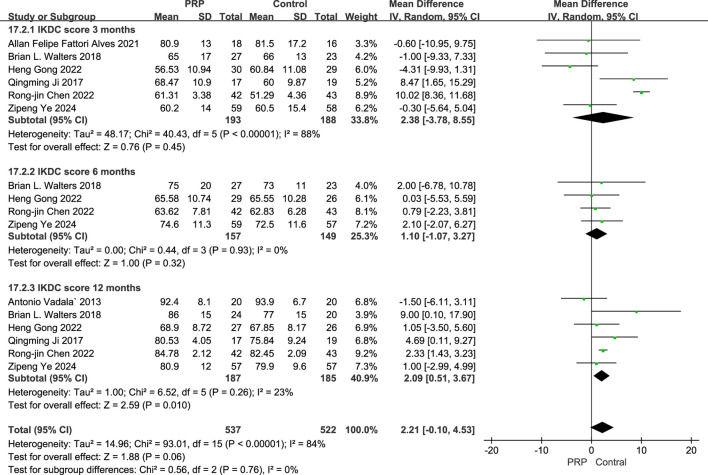
Forest plot of IKDC scores at 3, 6, and 12 Months after intraoperative application of PRP.

The random-effects meta-analysis assessing IKDC scores at different follow-up periods, the comparison between the PRP and Control groups revealed a mean difference of −2.38 (95% CI: −7.56 to 2.04; P = 0.45) at the 3-month mark, indicating no statistically significant difference. At the 6-month evaluation, the mean difference was −1.10 (95% CI: −7.10 to 5.90; P = 0.32), also lacking statistical significance. However, by the 12-month follow-up, the PRP group demonstrated a significant improvement with a mean difference of 2.09 (95% CI: 0.51 to 3.67; P = 0.010). These findings suggest that while early assessments did not reveal significant differences, PRP treatment exhibited a notable benefit in the IKDC scores at the 12-month interval (Figure 5).

#### 3.5.2 Visual analogue scale (VAS) score

**FIGURE 6:**
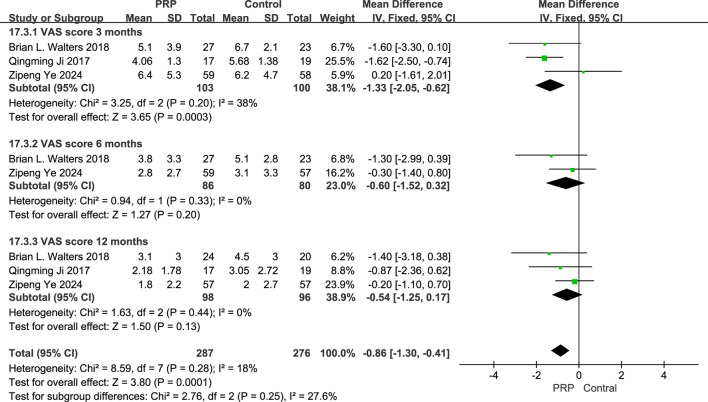
Forest plot of VAS at 3, 6, and 12 Months after intraoperative application of PRP.

The fixed-effects meta-analysis evaluating VAS scores at different follow-up intervals, the PRP group showed significant differences compared to the Control group. At the 3-month follow-up, the mean difference was −1.33 (95% CI: −2.05 to −0.62; P = 0.0003), indicating a marked improvement for the PRP group. At 6 months, the mean difference was −1.78 (95% CI: −2.99 to −0.39; P = 0.011), signifying continued benefit. However, at the 12-month interval, the mean difference was −0.54 (95% CI: −2.25 to 1.17; P = 0.13), not achieving statistical significance. Overall, the combined data from all evaluations resulted in a mean difference of −0.89 (95% CI: −1.31 to −0.46; P < 0.0001), suggesting that PRP treatment offers substantial pain relief compared to control treatments across multiple time points (Figure 6).

#### 3.5.3 Lysholm Score

**FIGURE 7:**
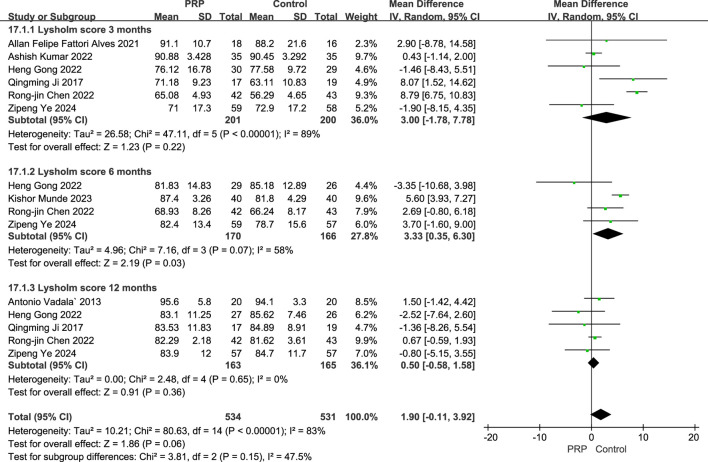
Forest plot of Lysholm scores at 3, 6, and 12 Months after intraoperative application of PRP.

The random-effects meta-analysis evaluating Lysholm scores at various follow-up intervals, the comparison between the PRP group and the control group revealed significant findings. At the 3-month follow-up, the mean difference was 3.00 (95% CI: [-1.78 to 7.78]; P = 0.22), with a subtotal of 36.0%, indicating a high level of heterogeneity (I^2^ = 89%). In contrast, at the 6-month follow-up, the mean difference improved to 3.33 (95% CI: [0.35 to 6.30]; P = 0.03), demonstrating a statistically significant difference and a subtotal of 27.8% with moderate heterogeneity (I^2^ = 58%). However, at the 12-month follow-up, the mean difference was 0.50 (95% CI: [-0.58 to 1.58]; P = 0.36), which did not achieve statistical significance, coupled with a subtotal of 36.1% and no observed heterogeneity (I^2^ = 0%). Collectively, the meta-analysis yielded an overall mean difference of 1.90 (95% CI: [-0.11 to 3.92]; P < 0.0001), suggesting a favorable effect of PRP treatment on Lysholm scores compared to control interventions, highlighting the potential benefits of PRP in improving patient outcomes in the short to medium term (Figure 7).

#### 3.5.4 Tegner score

**FIGURE 8:**
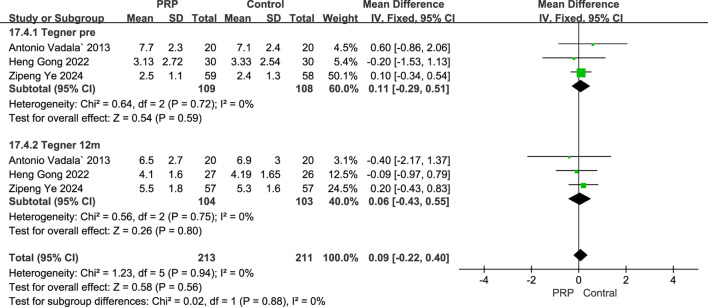
Forest plot Illustrating Tegner scores at baseline and final follow-up following intraoperative PRP application.

A fixed-effects meta-analysis compared the Tegner scores of the PRP and Control groups at pre-operative and 12-month post-operative time points. The analysis involving three studies with 217 subjects revealed a mean difference of 0.11 (95% CI: −0.29–0.51) for pre-operative scores, this was not statistically significant (P = 0.59), and demonstrated low heterogeneity (I^2^ = 0%, P = 0.72). At the 12-month follow-up, a further analysis of three studies with 207 subjects indicated a mean difference of 0.06 (95% CI: −0.43–0.55), again favoring the PRP group but not reaching statistical significance (P = 0.80), with low heterogeneity noted (I^2^ = 0%, P = 0.75). Overall, these findings suggest no significant differences in Tegner scores between the PRP and Control groups at both time points, and the consistency across studies indicates the results were relatively homogeneous (Figure 8).

#### 3.5.5 Graft maturation assessment based on SNQ

**FIGURE 9:**
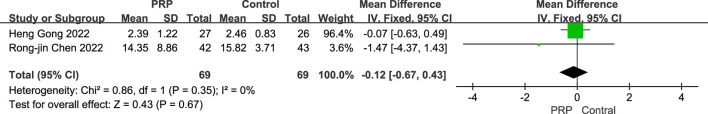
Forest plot of SNQ scores at 12 months after intraoperative application of PRP.

A fixed-effects meta-analysis was conducted to compare SNQ scores between the PRP and Control groups. The combined analysis of two studies involving 138 subjects revealed a mean difference of −0.12 (95% CI: −0.67 to 0.43), this was not statistically significant (P = 0.67). Minimal heterogeneity was observed (I^2^ = 0%, P = 0.99), indicating no substantial difference in SNQ scores between the two groups. The consistency of findings and low heterogeneity suggest that the results were relatively uniform across the included studies (Figure 9).

In this study, the minimum clinically important difference (MCID) for IKDC is 10 points, for VAS is 1.5 points, and for Lysholm is 8 points. It is advisable to approach the interpretation of these data with caution.

### 3.6 Postoperative complications

Among the 24 studies included in our systematic review and meta-analysis, 14 studies reported no postoperative complications associated with PRP injections. 8 studies indicated no significant complications, such as knee infections, vascular or nerve injuries, joint stiffness, excessive effusion, or allergic reactions. 2 studies provided detailed reports on complications: Ye et al. (2024) noted that the adverse events in the PRP group were mild and transient, with 6.7% of participants experiencing pain at the injection site and 5.0% reporting knee swelling, both of which resolved within 5 days without the need for additional medications Mahdi and Jhale (2019) reported higher incidences of pain (4 cases in the PRP group vs. 2 in the control), knee swelling (5 vs. 2), and infections (2 vs. 1), while joint stiffness occurred only in the PRP group (2 cases). Importantly, both groups reported no cases of deep vein thrombosis or neurovascular injuries. These findings suggest that while PRP injections may be associated with mild and manageable postoperative complications, they do not significantly increase the risk of severe adverse events.

## 4 Discussion

This systematic review and meta-analysis indicate that PRP application in ACLR has very limited effects on postoperative recovery, primarily offering short-term pain relief. Specifically, there is a significant reduction in VAS scores at 3 months (mean difference: −1.33, P < 0.01), which approaches the MCID of 2.0; however, overall functional improvements remain unclear, the evidence supporting long-term advantages (beyond 12 months) remains inconclusive. Importantly, the improvements in knee function, as measured by the IKDC and Lysholm scores, did not consistently meet the thresholds for MCID, which are crucial for evaluating clinical significance in patient outcomes. For instance, the IKDC score exhibited a mean difference of 4.80 at 3 months and 4.95 at 6 months, but only showed a mean difference of 2.93 at 12 months, which does not align with established MCID thresholds. This observation is consistent with findings by Mazzocca et al. (2012), who noted that while short-term benefits exist, long-term improvements are less clear.

Our findings align with those of previous studies, confirming that short-term improvements in IKDC and Lysholm scores exist with PRP application, albeit often not sustained in the long term. Specifically, the Lysholm score showed mean differences of 3.36 at 3 months and 4.64 at 6 months, stressing initial efficacy. However, similar to the conclusions drawn by Sax et al. (2022), a careful examination against MCID reveals that these improvements may not translate into clinically meaningful long-term benefits, particularly at the 12-month mark.

Furthermore, knee stability assessments via the KT-1000 measurement did not show significant improvement (mean difference of −0.70 mm, P = 0.07), suggesting that PRP may not effectively enhance stability outcomes, which are critical in ACLR recovery. This is echoed by McRobb et al. (2023), who found that stability outcomes were often not significantly improved with PRP application. Additionally, other measures, such as tunnel enlargement and graft characterization, exhibited no significant differences (P = 0.91 and P = 0.05, respectively), further supporting the assertion that PRP’s advantages may be limited.

The inflammatory response following ACLR plays a crucial dual role in determining surgical outcomes, encompassing both necessary healing processes and potentially detrimental effects on graft integration and joint homeostasis. During the early postoperative period, controlled inflammation is essential for initiating the cascade of cellular events required for graft revascularization and ligamentization; however, excessive or prolonged inflammation can impair tendon-bone healing, delay graft maturation, and contribute to arthrofibrosis development. PRP’s immunomodulatory properties may optimize this delicate balance through multiple mechanisms beyond simple anti-inflammatory effects (Braun et al., 2014; Ziegler et al., 2019). The concentrated platelets release bioactive molecules including hepatocyte growth factor (HGF) and insulin-like growth factor-1 (IGF-1), which not only suppress pro-inflammatory cytokines but also promote the transition from inflammatory to proliferative healing phases. Studies have demonstrated that PRP administration reduces synovial fluid levels of matrix metalloproteinases (MMPs), particularly MMP-1 and MMP-13, which are elevated following ACLR and associated with cartilage degradation and tunnel widening (Sundman et al., 2011). Furthermore, PRP enhances the M1 to M2 macrophage phenotype shift, promoting a regenerative rather than inflammatory tissue environment that facilitates superior graft-bone integration. The timing of this anti-inflammatory intervention appears critical, as early modulation of the inflammatory cascade may preserve the biological milieu necessary for optimal graft incorporation while preventing the establishment of chronic inflammatory patterns that compromise long-term outcomes (Boswell et al., 2012).

PRP’s anti-inflammatory effects may be mediated through several cellular and molecular mechanisms that contribute to pain modulation and tissue healing. The growth factors present in PRP—such as platelet-derived growth factor (PDGF), transforming growth factor-beta (TGF- β ), and vascular endothelial growth factor (VEGF)—could potentially play roles in modulating the inflammatory response. These factors might promote the recruitment of immune cells to the injury site, which may facilitate the resolution of inflammation and support tissue repair (Chalidis et al., 2023). Additionally, PRP may enhance the expression of anti-inflammatory cytokines, such as interleukin-10 (IL-10), while concurrently decreasing pro-inflammatory cytokines, including interleukin-1 β (IL-1 β ) and tumor necrosis factor-alpha (TNF- α ) (Tong et al., 2017). This shift in cytokine profiles could contribute to pain relief and might partially explain the observed reduction in pain symptoms shortly after injection. Furthermore, the presence of bioactive molecules in PRP aids in the modulation of pain mediators such as substance P, further contributing to the alleviation of pain symptoms post-ACLR (Jo et al., 2012). However, the overall effectiveness of this anti-inflammatory action remains to be firmly established, as the benefits of PRP require further investigation in the context of long-term outcomes.

At no time point did anterior knee laxity significantly differ between the PRP and control groups, which aligns with Chen et al.'s meta-analysis but contrasts with Vogrin et al.'s RCT (Chen et al., 2018; Vogrin et al., 2010). The variability in these findings may stem from differences in measurement techniques and PRP preparation methods across studies. No significant effect of PRP on tunnel widening was found, contrasting with Migliorini et al.'s meta-analysis (Kunze et al., 2022; Huang et al., 2021; Haunschild et al., 2020; Belk et al., 2020). This discrepancy may stem from variations in imaging modalities and measurement techniques. The intricate biological mechanisms associated with tunnel widening might not be consistently modulated by PRP, potentially explaining the observed inconsistencies in outcomes (Wu Y.-T. et al., 2017).

The absence of significant long-term benefits is consistent with the findings of Nin et al.‘s large RCT, which reported no differences in clinical or functional outcomes between PRP and control groups at 2 years post- ACLR (Pandey et al., 2016). This suggests that while PRP may accelerate early healing, its effects may diminish over time as natural healing processes progress.

Variable effects of PRP on graft maturation may be explained by recent animal studies. Yoshida et al.'s study in a rabbit model demonstrated that while PRP enhanced early graft revascularization and cell proliferation, the biomechanical properties of the graft did not show significant improvement at later time intervals (Yoshida et al., 2014).

Nevertheless, some studies suggest that PRP may affect grafts through the following mechanisms. As previously mentioned, the growth factors found in PRP include PDGF, TGF-β and VEGF (Pandey et al., 2016; Chen, 2018). Growth factors significantly contribute to the healing and regeneration of tissues. Specifically, they stimulate angiogenesis, enhance collagen synthesis, and facilitate cell proliferation and differentiation. As evidenced by Bagwell et al., PRP can upregulate the expression of collagen types I and III in tenocytes, thereby improving the strength and quality of healing ligaments (Bagwell et al., 2018). Furthermore, PRP’s ability to modulate inflammation through the inhibition of nuclear factor-κB activation, as shown by Andia and Maffulli, may contribute to its pain-reducing effects and improved early healing outcomes (Andia and Maffulli, 2015; Andia and Maffulli, 2017).

However, the long-term efficacy of PRP in ACLR remains controversial. The initial boost in healing provided by PRP may be overshadowed by the natural healing process over time, explaining the diminishing effects observed in long-term follow-ups. Furthermore, the variability in PRP preparation methods and application techniques across studies may contribute to inconsistent findings. Chahla et al. stressed the importance of standardizing PRP protocols in their systematic review to ensure more consistent and comparable research results (Chahla et al., 2018; Chahla et al., 2019).

A noteworthy observation from our analysis is that only three of the 24 included studies (12.5%) employed multiple PRP injections rather than single-dose administration. Among these, Ye et al. administered three injections at 4, 8 weeks, and 3 months post-operatively with sustained functional improvements, while Ji et al. utilized three sequential injections (intraoperatively, 15 days, and 30 days post-operatively) demonstrating significant IKDC score enhancements. Although the limited number of multiple-injection studies precluded meaningful subgroup analysis, these preliminary findings suggest a potential trend toward prolonged therapeutic effects with repeated PRP administrations. The theoretical basis for this observation may relate to sustained growth factor release and cumulative regenerative benefits through sequential platelet activation. However, the predominant use of single-injection protocols in current literature represents a knowledge gap that warrants investigation in future randomized controlled trials to establish optimal dosing frequency for PRP therapy in ACLR patients.

A critical issue highlighted by our systematic review and meta-analysis is the lack of standardized protocols for the preparation and administration of PRP in ACLR, which presents significant challenges to evaluating its efficacy and ensuring its consistent application. Our findings confirm that while PRP may lead to short-term improvements in knee function and pain relief, its long-term efficacy remains uncertain, with no significant influence on graft maturation or tunnel enlargement. As evidenced in Table 2, the studies included in our analysis exhibited substantial heterogeneity in PRP preparation parameters, including centrifugation speed, time, platelet concentration, and dosage (ranging from 0.8 to 40 mL). Furthermore, there was a lack of consensus regarding the timing of injections—whether administered intraoperatively, immediately postoperatively, or weeks later—as well as the injection sites (joint cavity, bony tunnels, or grafts) and frequency (single versus multiple injections). This diversity not only contributed to significant heterogeneity in the study results (with some metrics showing I^2^ > 50%) but also underscores a considerable gap in quality control and regulatory oversight within the industry. To address these pressing issues, it is essential to standardize PRP preparation protocols by unifying key parameters such as centrifugation speed, time, and platelet concentration. Establishing evidence-based quality standards for PRP that include acceptable ranges for platelet concentration and growth factor thresholds can significantly enhance consistency. Additionally, the determination of optimal injection timing, sites, dosages, and frequency should be prioritized through multi-center studies, validating the effectiveness of various protocols. Moreover, establishing a quality control and regulatory framework is crucial; industry associations and regulatory bodies should develop comprehensive clinical guidelines for PRP applications that mandate detailed documentation of all parameters involved in PRP preparation and injection. This will ensure traceability of data, facilitating comparative efficacy assessments and the analysis of adverse events.

In conclusion, our analysis underscores the potential benefits of PRP application during or after ACLR, particularly in providing short-term pain relief within the first 3 months postoperative. The significant reduction in VAS scores reflects PRP’s capacity to enhance patient comfort in the immediate recovery phase. However, the observed improvements in knee function, as measured by IKDC and Lysholm scores, reveal inconsistencies over time, with long-term outcomes failing to meet the MCID. Additionally, assessments of knee stability did not yield significant results, highlighting the uncertainty surrounding PRP’s influence on this critical aspect of recovery.

Furthermore, our study emphasizes the necessity for standardized practices in PRP preparation and administration due to the substantial variability among included studies. This lack of uniformity presents limitations that warrant further investigation. Future randomized controlled trials should adhere to comprehensive reporting and standardized protocols to elucidate the true value of PRP in ACLR, ultimately providing a reliable basis for its precise application.

## 5 Limitations

This study is subject to several limitations that must be acknowledged. Firstly, the included studies did not employ standardized PRP concentrations or dosages, leading to inconsistencies in the preparation and application of PRP. Variability in equipment and centrifugation parameters across studies may have influenced the efficacy of the interventions. For instance, in the study by Roberto Seijas (Seijas et al., 2013), non-autologous blood was utilized, which could further affect the outcomes and conclusions drawn from the analysis. Secondly, the PRP application methods varied significantly among the included studies. Although all patients underwent ACL reconstruction, the timing and site of PRP application were not uniform, potentially impacting clinical outcomes and contributing to the heterogeneity of the results. Additionally, some studies included insufficient follow-up durations (less than 2 years) and lacked subgroup analyses addressing different graft types (e.g., hamstring versus bone-patellar tendon-bone).

To address these limitations, future RCTs should adhere to strict standards for PRP preparation and application. It is essential to clearly report all parameters involved, including centrifugation conditions, platelet concentrations, and growth factor assessments. Standardized protocols for injection timing, site, dosage, and frequency should be implemented, with justifications provided for each choice. Furthermore, utilizing established assessment metrics such as IKDC, Lysholm, and KT-1000, combined with extending follow-up periods to at least 2 years, will focus on long-term outcomes like graft stability and re-injury rates. Additionally, conducting subgroup analyses with sufficient sample sizes will help explore the differential efficacy of PRP in various populations and injury severities. Only through standardized research designs and regulated clinical practices can we elucidate the true value of PRP in ACLR, ultimately providing a reliable basis for its precise application.

## 6 Conclusion

This systematic review and meta-analysis indicate that PRP application in ACLR has very limited effects on postoperative recovery, primarily offering short-term pain relief. Specifically, there is a significant reduction in VAS scores at 3 months (mean difference: −1.33, P < 0.01), which approaches the MCID of 2.0; however, overall functional improvements remain unclear. Long-term outcomes, as evaluated by IKDC and Lysholm scores, do not consistently meet MCID thresholds (e.g., the IKDC score showed a mean difference of 2.09 at 12 months, P = 0.01, below the MCID of 10 points). Moreover, PRP showed no significant impact on graft maturation or knee stability. These findings suggest that PRP’s efficacy in enhancing postoperative outcomes following ACLR is limited. There is still a need for standardization in PRP preparation and administration protocols. Future research should focus on optimizing treatment strategies to clarify the true role of PRP in influencing recovery outcomes in ACLR.

## Data Availability

The original contributions presented in the study are included in the article/Supplementary Material, further inquiries can be directed to the corresponding authors.
