# Supplementary material for: The impact of platelet-rich plasma injection on anterior cruciate ligament reconstruction: a systematic review and meta-analysis
Source: Front Bioeng Biotechnol. 2025 Oct 1;13:1625271. doi: 10.3389/fbioe.2025.1625271 (PMC12521423; doi:10.3389/fbioe.2025.1625271)
Supplement: Supplementary file 1 [file DataSheet1.pdf]

Supplementary Appendix

Title: **The Impact of Platelet-Rich Plasma Injection on Anterior Cruciate Ligament Reconstruction: A Systematic Review and Meta-Analysis**

This supplementary appendix provides the complete search strings used in various databases to enhance transparency and replicability of our study.

Database Search Strategies

1. PubMed

No.	Query	Results
#1	((((((((((((((((((((((((((Anterior Cruciate Ligament Reconstruction[MeSH Terms]) OR (Anterior Cruciate Ligament Injuries[MeSH Terms])) OR (Anterior Cruciate Ligament Tears[MeSH Terms])) OR (Anterior Cruciate Ligament Tear[MeSH Terms])) OR (Anterior Cruciate Ligament Injury[MeSH Terms])) OR (ACL Tear[MeSH Terms])) OR (ACL Tears[MeSH Terms])) OR (Tear, ACL[MeSH Terms])) OR (Tears, ACL[MeSH Terms])) OR (Injury, ACL[MeSH Terms])) OR (ACL Injury[MeSH Terms])) OR (Injuries, ACL[MeSH Terms])) OR (ACL Injuries[MeSH Terms])) OR (Anterior Cruciate Ligament[MeSH Terms])) OR (Anterior Cranial Cruciate Ligament[MeSH Terms])) OR (Cranial Cruciate Ligament[MeSH Terms])) OR (Cruciate Ligaments, Cranial[MeSH Terms])) OR (Cranial Cruciate Ligaments[MeSH Terms])) OR (Ligaments, Cranial Cruciate[MeSH Terms])) OR (Cruciate Ligament, Cranial[MeSH Terms])) OR (Ligament, Cranial Cruciate[MeSH Terms])) OR (Ligament, Anterior Cruciate[MeSH Terms])) OR (Cruciate Ligaments, Anterior[MeSH Terms])) OR (Ligaments, Anterior Cruciate[MeSH Terms])) OR (Anterior Cruciate Ligaments[MeSH Terms])) OR (Cruciate Ligament, Anterior[MeSH Terms])) AND (((((Platelet-Rich Plasma[Title/Abstract]) OR (Platelet Rich Plasma[Title/Abstract])) OR (Plasma, Platelet-Rich[Title/Abstract])) OR (Thrombocyte Rich Plasma[Title/Abstract])) OR (PRP[Title/Abstract]))	117

2. Web of Science

No.	Query	Results
#1	"((((((ALL=(Platelet-Rich Plasma)) OR ALL=(Thrombocyte Rich Plasma)) OR ALL=(PRP)) OR ALL=(platelet-rich plasma cell)) OR ALL=(platelet rich plasma cell)) OR ALL=(Platelet Rich Plasma)) OR ALL=(Plasma, Platelet-Rich)	38644
#2	"(((((((((((((((((((ALL=(Anterior Cruciate Ligament Injuries)) OR ALL=(Anterior Cruciate Ligament Reconstruction)) OR ALL=(Anterior Cruciate Ligament)) OR ALL=(Anterior Cruciate Ligament Tears)) OR ALL=(Anterior Cranial Cruciate Ligament)) OR ALL=(Cranial Cruciate Ligament)) OR ALL=(Cruciate Ligaments, Cranial)) OR ALL=(Cranial Cruciate Ligaments)) OR ALL=(Ligaments, Cranial Cruciate)) OR ALL=(Cruciate Ligament, Cranial)) OR ALL=(Ligament, Cranial Cruciate)) OR ALL=(Ligament, Anterior Cruciate)) OR ALL=(Cruciate Ligaments, Anterior)) OR ALL=(Ligaments, Anterior Cruciate)) OR ALL=(Anterior Cruciate Ligaments)) OR ALL=(Cruciate Ligament, Anterior)) OR ALL=(ACL Tear)) OR ALL=(ACL Tears)) OR ALL=(ACL Injury)) OR ALL=(Injuries, ACL)) OR ALL=(ACL Injuries) "	34905
#3	#1 AND #2	409

3. Embase

No.	Query	Results
#1	'anterior cruciate ligament reconstruction'/exp OR 'anterior cruciate ligament reconstruction'/syn	22129
#2	'platelet-rich plasma cell'/exp OR 'platelet-rich plasma cell'/syn OR 'anterior cruciate ligament reconstruction'/syn	55982
#3	#1 AND #2	150

Last Search Dates
PubMed: 7-Jul-24
Web of Science: 7-Jul-24
Cochrane Library: 7-Jul-24

Eligible Studies
A total of 676 eligible studies were identified from the searches across the databases.
